# The General Self-Efficacy of Older Adults Receiving Care: A Systematic Review and Meta-Analysis

**DOI:** 10.1093/geront/gnaa036

**Published:** 2020-05-06

**Authors:** Lucy Whitehall, Robert Rush, Sylwia Górska, Kirsty Forsyth

**Affiliations:** School of Health Sciences, Queen Margaret University, Edinburgh, UK

**Keywords:** Analysis—systematic review, Analysis—meta-analysis, Hospital/ambulatory care, Nursing homes, Home- and community-based care and services, Rehabilitation, Autonomy and self-efficacy

## Abstract

**Background and Objectives:**

General self-efficacy (GSE) encourages health-promoting behaviors in older adults. It is unsurprising then that older adults receiving health care services are reported to have a greater risk of low GSE than older adults who are not. Despite this, there is currently limited evidence investigating whether the effect differs based on the environment in which care is received. This review aims to determine whether the GSE of older adults is affected by the receipt of health care services and whether GSE varies based on the setting in which care is received.

**Research Design and Methods:**

In accordance with PRISMA guidelines (PROSPERO registration number CRD42018092191), a systematic search was undertaken across 7 databases. Standardized mean differences (SMD) and mean General Self-Efficacy Scale scores, with 95% confidence intervals (CI), were pooled for meta-analysis.

**Results:**

A total of 40 studies were identified, they consisted of 33 population cohorts that were included in the meta-analysis. Older adults receiving health care services were found to be at greater risk of having lower GSE than those who do not (SMD = −0.62; 95% CI: −0.96 to −0.27, *p* < .0001). Following identification of sources of heterogeneity, older adults receiving acute inpatient care were more likely to have lower GSE than those receiving care in other health care settings.

**Discussion and Implications:**

Older adults receiving inpatient care have a greater risk of lower GSE, and consequently, poorer health-promoting behaviors. Further research is recommended that focuses on the GSE of older adults and health outcomes following discharge from inpatient care.

## Background

Advances in medical care and public health mean that the world’s older population is growing; between 2025 and 2050, the global population of adults aged 65 and over is predicted to almost double to 1.6 billion (United Nations, 2015).

With increasing age comes increasing multimorbidity and functional dependency, and the complex care required to manage these often increases health service use (He, Goodkind, & Kowal, 2015). As a result, the most frequent users of health care services are individuals aged between 75 and 80 years (Chawla, Betcherman, & Banerji, 2007; Peltzer et al., 2014). As the world’s older population grows, health care utilization is increasing too, and is contributing to increasing health care expenditure (He, Goodkind, & Kowal, 2015; Rechel, Doyle, Grundy, & Mckee, 2009).

However, current evidence suggests that through extending the healthy life expectancy of older people, lifetime health care expenditures may be reduced (Fried, 2011; He et al., 2015; Suhrcke, Arce, McKee, & Rocco, 2008).

Contemporary conceptual models of healthy aging are built around the functional ability of older people to participate in meaningful activities, promoting quality of life, and reducing dependency, rather than around the absence of disease. In order for health systems to adapt to population aging, public policy needs to adopt these models to support healthy aging, thus reducing the use of health care services and easing the financial pressures on health care systems (Rechel et al., 2009).

The ability of health care professionals to support healthy aging and extend the healthy life expectancy of older adult populations requires the identification of factors that indicate poor health-promoting behaviors. Correspondingly, there has been increasing focus on the role of positive psychological resources, which are expected to play a role in reducing suffering on the health of older adults (Santo & Daniel, 2018).

This field of research has frequently investigated the relationship between the health-promoting behaviors of older adults and general self-efficacy (GSE), which explains how individuals cope with daily struggles and adapt to stressful life events (Schwarzer, 1992). GSE is understood to be an operative construct, that is, it is related to subsequent behavior and, therefore, is relevant for clinical practice (Jones, Mandy, & Partridge, 2009; Schwarzer, 1992; Tousignant et al., 2012).

Older adults with lesser GSE have consistently been found to limit their involvement in activities of daily living (ADL) and reduce their efforts in activities they do complete (Easom, 2003). In contrast, those with a greater level of GSE are more proactive in seeking health information, engaging in self-care behaviors, making health modifications, and adhering to treatment (Easom, 2003; Kostka & Jachimowicz, 2010; Rodin, 1986; Stadtlander, Giles, & Sickel, 2015). Concerning health care use, increased health problems and increased contact with health care services may undermine older adults’ GSE (Rodin, 1986). Moreover, it is suggested that the frequency or length of contact with the health care services may heighten these restrictions (Bandura, 1982; Woodward & Wallston, 1987). It is, therefore, of importance that GSE is considered and addressed by the health care services that aim to promote well-being and independence.

Current research has investigated the GSE of older adults receiving different levels and forms of health care provision in order to ascertain whether GSE interventions may improve the quality of life and healthy aging of older adults in the face of ill health (Bonsaksen, Lerdal, & Fagermoen, 2012; Cybulski, Cybulski, Krajewska-Kulak, & Cwalina, 2017; Kostka & Jachimowicz, 2010; Mystakidou et al., 2015). However, very little research has investigated whether there is any effect of the health care setting on the GSE of older adults, despite it being understood that the design of care settings may influence a range of patient health outcomes (Ulrich, Zimring, & Zhu, 2008).

Only one study has investigated the difference in levels of GSE between older adult populations receiving care in different health care settings. This study suggested that the form of health care an older adult receives may influence their level of GSE, with participants receiving acute inpatient care having lower GSE than individuals receiving rehabilitative or long-term care (Barder, Slimmer, & LeSage, 1994). While no further studies have assessed the GSE of older adults across multiple care settings, more recent research has investigated the difference in the GSE between populations of “healthy older adults” and “older adults receiving care.” They shared the same intention of identifying whether specific populations have lower GSE and should be the first focus of intervention (Cybulski et al., 2017; Kim, Jeon, Sok, & Kim, 2006; Kostka & Jachimowicz, 2010; Schmidt, Wahl, & Plischke, 2014).

As GSE is understood to impact upon older adults’ participation in ADL, their abilities to make health modifications and adjust to ill health (Easom, 2003; Kostka & Jachimowicz, 2010; Rodin, 1986; Stadtlander, Giles, & Sickel, 2015), interventions focused on enhancing older adults’ GSE have been identified as having the potential to develop clinical practice and improve patient health outcomes.

However, it is recognized that GSE may be altered by the receipt of health care services and the environment in which they are received (Rodin, 1986; Ulrich et al., 2008). Previous research has focused on the effectiveness of GSE interventions; however, little attention has been paid to the difference in GSE between older adult populations receiving care in different health care settings.

In recognition of this, we conducted a systematic review and meta-analysis, using current evidence. We aimed to determine whether older adults’ who receive health care services have lower GSE than those who do not, and to investigate whether older adults’ receiving health care services are at risk of having lower GSE based on the environment in which care is received.

## Methods

### Protocol and Registration

This systematic review and meta-analysis were conducted in accordance with PRISMA guidelines (see Supplementary Material; Knobloch, Yoon, & Vogt, 2011). A review protocol was published with PROSPERO (registration number CRD42018092191).

### Eligibility Criteria

This review included both observational and interventional study designs, providing they presented the mean score and standard deviation of the GSE scale used.

Participants were required to be “receiving care at the time of assessment.” Decisions as to whether studies met this criterion were made by two members of the review team in consideration of the purpose of the study, the study procedure, and information given regarding the participants.

The most recent findings of the European Social Survey (Abrams, Russell, Vauclair, & Swift, 2011) found that the average perceived start of old age was 62 years (range: 55.1–68.2). Accordingly, each study population included in this review had to have a lower 95% confidence interval (CI) of at least 60 years old. No exclusion criteria limited the participants by gender, clinical diagnosis, length of care, or the type of care being received, assuming it was reported and could be categorized into “inpatient care,” “outpatient care,” or “community care.”

Throughout GSE research, three GSE measures are routinely used. These are the Generalized Self-Efficacy Scale (GSES) (Schwarzer & Jerusalem, 1995), the 17-item, five-point scale, GSE section of the Self-Efficacy Scale (SES) (Sherer & Adams, 1983; Sherer et al. 1982), and the New General Self-Efficacy Scale (NGSES) (Chen, Gully, & Eden, 2001). All three of these tools demonstrate appreciable relationships with the latent construct of GSE (Scherbaum, Cohen-Charash, & Kern, 2006). Thus, studies that used one of these three measures were eligible for inclusion.

Finally, eligible studies had to be published in peer-reviewed journals and written in English.

### Search Strategy

In September 2019, searches were conducted on MEDLINE (EBSCOhost), PsycINFO (EBSCOhost), CENTRAL (Cochrane Library), CINAHL (EBSCOhost), Scopus (Elsevier), Abstracts in Social Gerontology (EBSCOhost), and ASSIA (ProQuest).

Keywords followed the PICOS principles (see Supplementary Material for detailed search strategy), including:

Population: elder* or “elderly people” or “older adults” or “older people” or aged or “aged, 80 and over” or geriatric*

Intervention: “hospital” or “nursing home” or “institutionaliz*” or “rehabilitation”

Outcomes: “self-efficacy” or “self-efficacy” or “efficacy beliefs” or “control” or “subjective wellbeing.”

Search terms were broad because narrowing them further resulted in eligible studies not being identified. This was primarily because titles and abstracts would either refer to participants’ specific health condition rather than where their care was received, or they would state they measured “self-efficacy” but not the tool used.

It is difficult to generalize gerontological research conducted several decades ago to a population of today’s older adults due to consecutive generations of older adults appearing strikingly different (Pew Research Centre, 2015; Rodin, 1986). Consequently, a date restriction of post-2000 was applied. Inspection of search results revealed that 95% of original studies were returned following restriction. Reference searches were conducted on studies eligible after full-text screening.

### Study Selection

Titles and abstracts were screened for appropriateness (L. Whitehall); studies not meeting the previously defined selection criteria were eliminated. Quasirandom sampling, based on the first author’s surname, was used to select 25% of the titles and abstracts, which were screened by a second member of the review team (N.D.) to prevent errors in methodology and reduce risk of bias.

The full text of remaining studies was retrieved and the decision to include in the review was made by the primary author and a second member of the review team (L.W. and N.D.). Disagreements regarding study eligibility were resolved through discussion with a third member (S.T.). Where decisions regarding eligibility were affected by missing data, attempts were made to contact the authors for clarification.

### Risk of Bias Assessment

Quality assessment of studies should use tools specific to their study designs (Harrison, Reid, Quinn, & Shenkin, 2017). As such, the included studies were assessed for bias using the appraisal instruments outlined in Table 1.

**Table 1.** Risk of Bias Assessment Instruments for Included Studies

Study design	Assessment instrument
Cross-sectional	Quality Assessment Tool for Observational Cohort and Cross-Sectional Studies (National Heart, Lung, and Blood Institute [NIH], 2014b)
Observational cohort	
Before–after with no control group	Quality Assessment Tool for Before–After (Pre–Post) Studies with No Control Group (NIH, 2014a)
Randomized controlled trial	Risk of Bias Tool (Higgins et al. 2011)
Controlled before–after	Risk of Bias Tool (Higgins et al. 2011)
Mixed methods	Mixed Methods Appraisal Tool (Pluye et al., 2011)
Secondary analysis of existing data	The REporting of studies Conducted using Observational Routinely collected health Data (RECORD) Statement (Benchimol et al., 2015)

Studies were classified as having high, moderate, or low risk of bias, in relation to their respective study designs. This classification is included in Table 2. For each study, risk of bias was assessed by the primary author and a second member of the review team (N.D.), based on instrument guidelines.

**Table 2.** Characteristics of the 40 Included Studies

Study	Country	Care setting	Diagnosis	Number of participants who completed GSE measure	% of males	Age (*SD*) (years)	Self-efficacy measure/baseline score	Risk of bias
Barnes-Harris et al. (2019)	United Kingdom and Australia	Outpatient clinics	Chronic breathlessness	41	59	70.5 (5.17)	Secondary data analysis of Johnson et al. (2016) and Swan et al. (2019)	Moderate
Bonsaksen et al. (2012)	Norway	Patient education course	Chronic obstructive pulmonary disease (COPD)	86	53	64.4 (9.7)	GSES 27.6 (6.4)	Moderate
Bonsaksen et al. (2013)	Norway	Patient education course	COPD	60	53	64.5 (9.4)	Same data as Bonsaksen et al. (2012)	Moderate
Bonsaksen et al. (2014)	Norway	Patient education course	COPD Obese group excluded due to age	56	58.9	66.3 (9.1)	Same data as Bonsaksen et al. (2012)	Low
Carlstedt et al. (2015)	Sweden	Outpatient clinic	Stroke	34	61.8	68.1	GSES 31.7 (6.95)	Moderate
Curtis, Groarke, and Sullivan (2014)	Ireland	Rapid access prostate hospital clinic	Prostate cancer	89	100	64.62 (8.02)	GSES 30.6 (5.64)	Low
Cybulski et al. (2017)	Poland	Nursing home	—	300 (nursing home = 100)	29 (overall)	60+	GSES NH: 24.38 (7.67) Community: 29.71 (3.88)	Low
Fan and Lv (2016)	China	Acute hospital cardiovascular wards	Chronic heart failure	159	47	63 (13.5)	GSES 26.11 (6.459)	Low
Feldstain et al. (2016)	Canada	Outpatient palliative rehabilitation	Stage 3/4 cancer	80	47.5	64.04 (12.5)	GSES 27.86 (6.16)	Low
Fors et al. (2018)	Sweden	Acute inpatient hospital	COPD and/or chronic heart failure	221 (intervention group = 103)	50.5 (intervention) 41.5 (control)	78.3 (9.5) (intervention) 76.9 (8.3) (control)	GSES Intervention: 28.1 (6.5) Control: 28.5 (5.8)	Unclear
Fu et al. (2018)	China	Nursing homes	—	307	29.3	60+	GSES 27.93 (5.6)	Low
Ghielen et al. (2017)	Netherlands	Outpatient clinic	Parkinson’s disease	19	55	66.6 (8.4)	GSES 28.5 (4.56)	Low
Haugland et al. (2016)	Norway	NET centers (outpatient)	Neuroendocrine tumors	196	49.5	65 (11)	GSES 29.9 (5.5)	Low
Huygens et al. (2017)	Netherlands	General practices	Chronic illness	627	49.9	65.1 (11.6)	GSES 3.12 (0.6)	Low
Iannello et al. (2018)	Italy	Inpatient rehabilitation	Hip fracture	42 (intervention group = 21)	28.5	79.95 (8.93) (intervention) 79.31 (9.12) (control)	GSES Intervention: 34.1 (7.5) Control: 32.7 (7.4)	Unclear
Johnson et al. (2016)	United Kingdom and Australia	Outpatient clinics	Refractory breathlessness	49 (intervention group = 24)	50 (intervention) 56 (control)	68.5 (11.6) (intervention) 67.7 (8.7) (control)	GSES Intervention: 30.2 (5.8) Control: 32.0 (4.4)	Low
Kim et al. (2006)	Korea	“Institutionalized”	—	214 (institutionalized = 106)	54.7 (institutionalized) 41.7 (noninstitutionalized)	65+	SES Institutionalized: 3.01 (0.44) Noninstitutionalized: 3.29 (0.42)	Low
Kosmat and Vranic (2017)	Croatia	Residential care home	—	22 (intervention group = 12)	25 overall	80.08 (6.156) (intervention) 79.08 (3.615) (control)	GSES Intervention: 3.3 (0.44) Control: 3.1 (0.56)	Unclear
Kostka and Jachimowicz (2010)	Poland	Nursing home	—	224 (nursing home = 112)	22.3 (nursing home) 21.5 (veterans’ home) 23.6 (community)	78.9 (6.9) (NH) 77 (7.6) (veterans’ home) 69.6 (4.6) (community)	GSES NH: 30.4 (7) Community: 30.9 (5.5)	Low
Lai et al. (2018)	Hong Kong	Nursing homes	—	96	34.4	84.6 (7.24)	GSES 21.55 (7.47)	Moderate
Lewin, Jöbges, & Werheid (2013)	Germany	Inpatient neurological rehabilitation	Stroke	96	52	67.08 (10.55)	Same data as Volz et al. 2018	Moderate
Liu et al. (2018)	China	Inpatient neurology wards	Ischemic stroke	147	63.3	68.57 (6.73)	GSES 25.6 (5.8)	Low
Magklara and Morrison (2016)	United Kingdom	Presurgery educational clinic	Prejoint replacement	53	33.3	69.33 (8.57)	GSES 31.19 (5.2)	Moderate
Mystakidou, Parpa, Tsilika, Galanos, & Vlahos (2008)	Greece	Inpatient palliative care unit	Palliative cancer	99	40.4	63.1 (14)	Same data as Mystakidou, Tsilika, et al. 2010	Low
Mystakidou, Tsilika, et al. (2010)	Greece	Inpatient palliative care unit	Palliative cancer	99	40.4	63.5 (13.2)	GSES 28.29 (6.9)	Low
Mystakidou, Parpa, et al. (2010)	Greece	Outpatient radiotherapy	Cancer	90 (male = 41) Female group excluded due to age	45.6	65.63 (12.48) (male) 57.45 (13.83) (female)	GSES Males: 33.17 (4.9)	Low
Mystakidou et al. (2012)	Greece	Outpatient palliative care	Cancer chronically ill group excluded due to age	107	51.4	64.52 (12.84)	GSES Cancer: 25.73 (6.0)	Low
Mystakidou et al. (2013)	Greece	Outpatient radiotherapy	Cancer	90	45.6	61.17 (5.2)	Same data as Mystakidou, Parpa, et al. 2010	Low
Mystakidou et al. (2015)	Greece	Outpatient palliative care	Palliative cancer	115	52.2	64.84 (12.7)	GSES 26.01 (6.11)	Low
Neuman et al. (2019)	United States	Inpatient hospital ward	Hip fracture	20	25	72 (10.96)	GSES 32.8 (6.2)	Moderate
Rotenberg Shpigelman, Sternberg, & Maeir (2019)	Israel	Geriatric clinics	Subjective memory complaints	91 (receiving care = 51)	45.1 (care receivers) 32.5 (not receiving care)	79.98 (7.33) (care receivers) 78.28 (7.4) (not receiving care)	NGSES Care receivers: 23.4 (6.1) Not receiving care: 26.4 (3.7)	Low
Schmidt et al. (2014)	Germany	Outpatient memory clinic	Mild cognitive impairment (MCI)	53 (MCI = 26)	46.7 (MCI) 47 (cognitively healthy)	72.5 (5.8) (MCI) 73.1 (7.0) (cognitively healthy)	GSES MCI: 28.5 (5.1) Cognitively healthy: 33.7 (5)	Low
Schulz et al. (2015)	Germany	Primary care	Osteoarthritis	1,018	31.1	74.9 (5.17)	GSES 32.7 (5.9)	Moderate
Stadtlander et al. (2015)	United States	Primary health care	—	35	17.1	88.4 (3.12)	GSES 31.3 (4.6)	Moderate
Strupeit et al. (2013)	Germany	Inpatient geriatric Rehabilitation	Mobility impairment	124 (intervention group = 39)	87.2 (intervention) 32.9 (control)	83.72 (6.87) (intervention) 83.44 (8.71) (control)	GSES Intervention: 25.97 (5.13) Control: 26.1 (6.25)	Moderate
Susanto et al. (2019)	Indonesia	“Institutional care”	Hypertension	64	59.4	71.86 (9.94)	GSES 27.88 (6.59)	Moderate
Swan et al. (2019)	United Kingdom	Outpatient clinics	Chronic breathlessness	40	70	72 (9.8)	GSES 31.03 (5.85)	Low
Tousignant et al. (2012)	Canada	Geriatric Day hospital	Falls	152 (intervention group = 76)	25 (intervention) 29 (control)	79.1 (6.4) (intervention) 80.7(6.0) (control)	GSES Intervention: 27.7 (6.2) Control: 29.2 (5.4)	Low
Volz et al. (2016)	Germany	Neurological inpatient rehabilitation	Stroke	88	54.5	66.35 (10.7)	Same data as Volz et al. 2018	Low
Volz et al. (2018)	Germany	Neurological inpatient rehabilitation	Stroke	294	58.9	63.78 (10.83)	GSES 31.25 (5.98)	Low

*Note*: GSE = General self-efficacy; GSES = Generalized Self-Efficacy Scale; SES = Self-Efficacy Scale; NH = nursing home; COPD = chronic obstructive pulmonary disease; MCI = mild cognitive impairment.

Funnel plots of publication bias were not created due to the expected heterogeneity resulting from the descriptive, observational nature of most studies (Terrin, Schmid, Lau, & Olkin, 2003).

### Data Extraction

The primary author extracted data using a prepiloted form adapted from the Joanna Briggs Extraction Form for Experimental and Observation Studies (Joanna Briggs Institute, 2014). This form comprised of four sections: general information, study design, participant characteristics, and general self-efficacy measure and score. A second member of the review team reviewed each completed form.

### Data Synthesis

Information regarding each study characteristic was extracted and is shown in Table 2.

Studies that compared the GSE between older adults receiving care versus noncare were meta-analyzed in Stata (StataCorp, 2019) using standardized mean differences (SMD; Figure 2).

**Figure 1.**
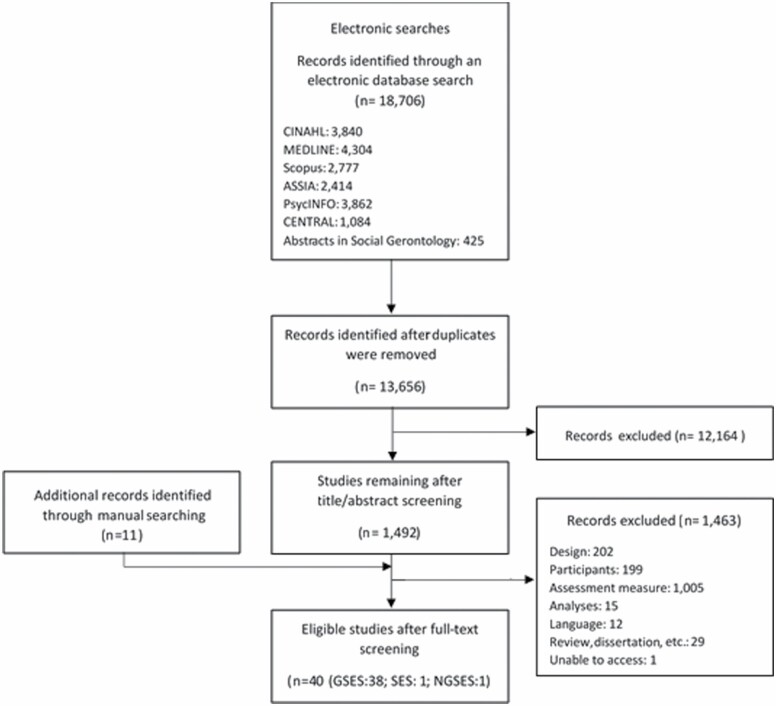
PRISMA diagram for study selection. GSES = Generalized Self-Efficacy Scale; NGSES = New General Self-Efficacy Scale; SES = Self-Efficacy Scale.

**Figure 2.**
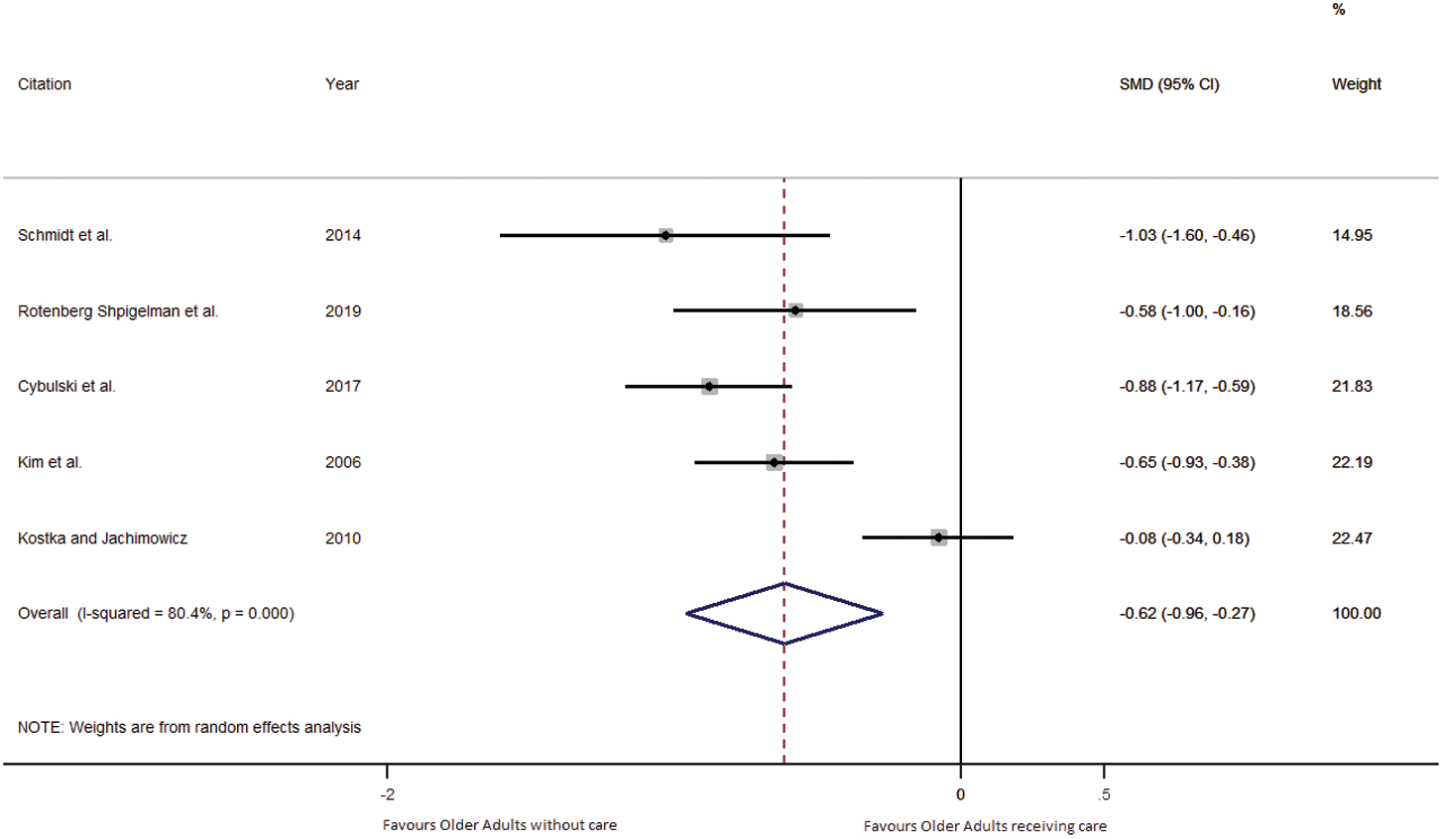
Forest plot demonstrating significantly lower GSE in older adults receiving care relative to older adults who are not receiving care. CI = confidence interval; GSE = General self-efficacy; SMD = standardized mean differences.

**Figure 3.**
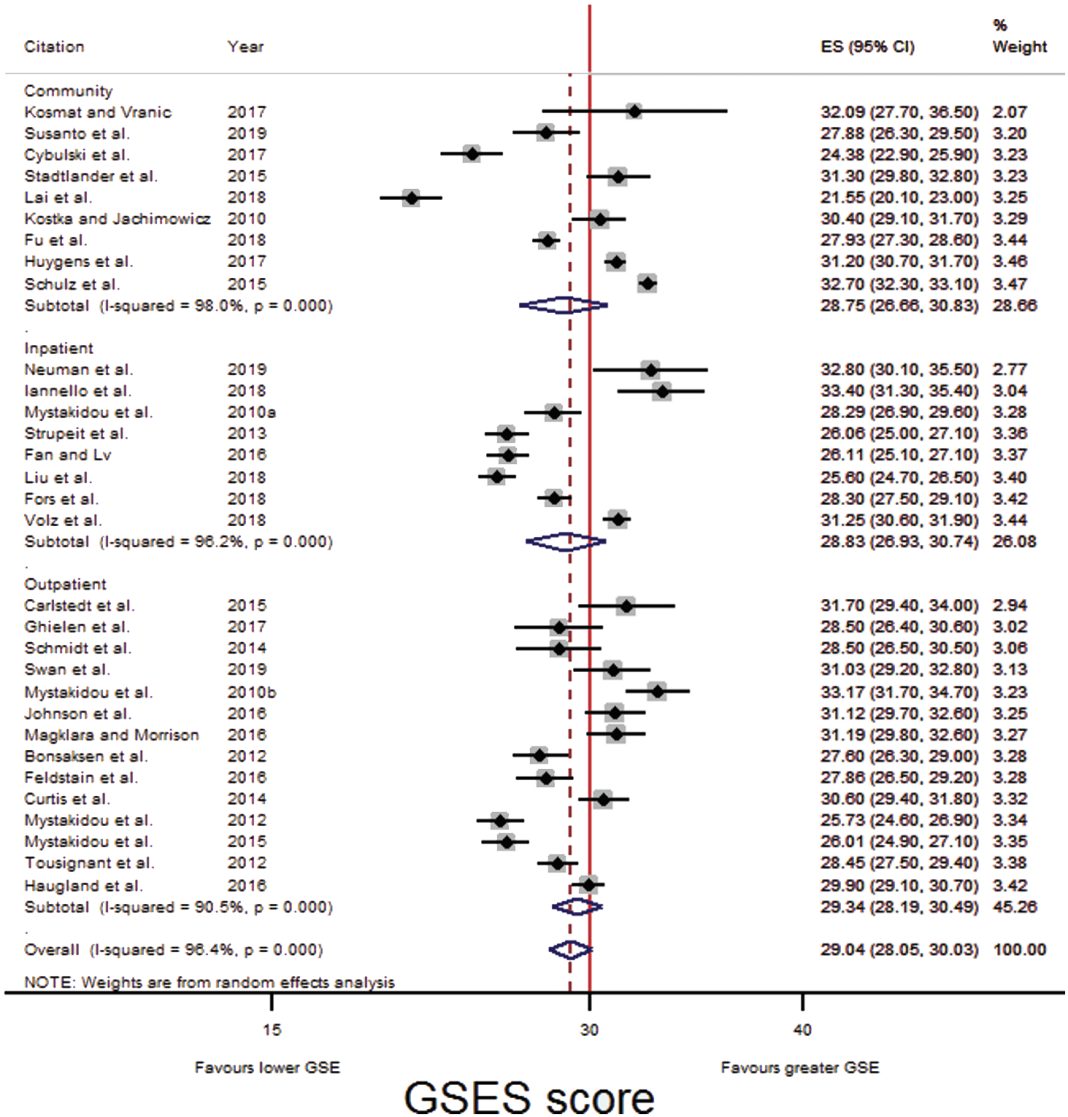
Forest plot: Comparison of GSES scores across three care settings. CI = confidence interval; GSES = Generalized Self-Efficacy Scale.

**Figure 4.**
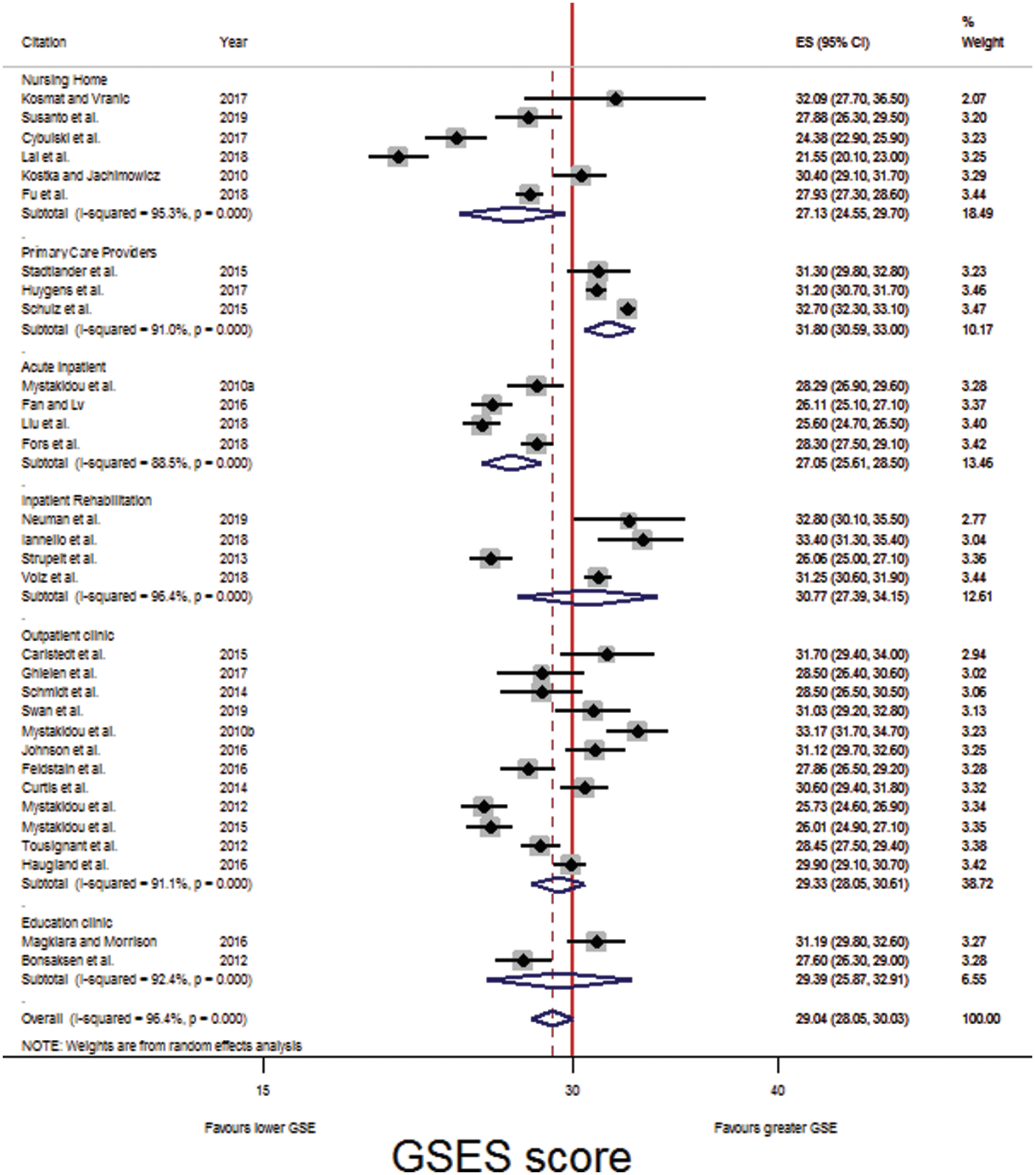
Forest plot: Comparison of GSES scores across six care settings. CI = confidence interval; GSES = Generalized Self-Efficacy Scale.

**Figure 5.**
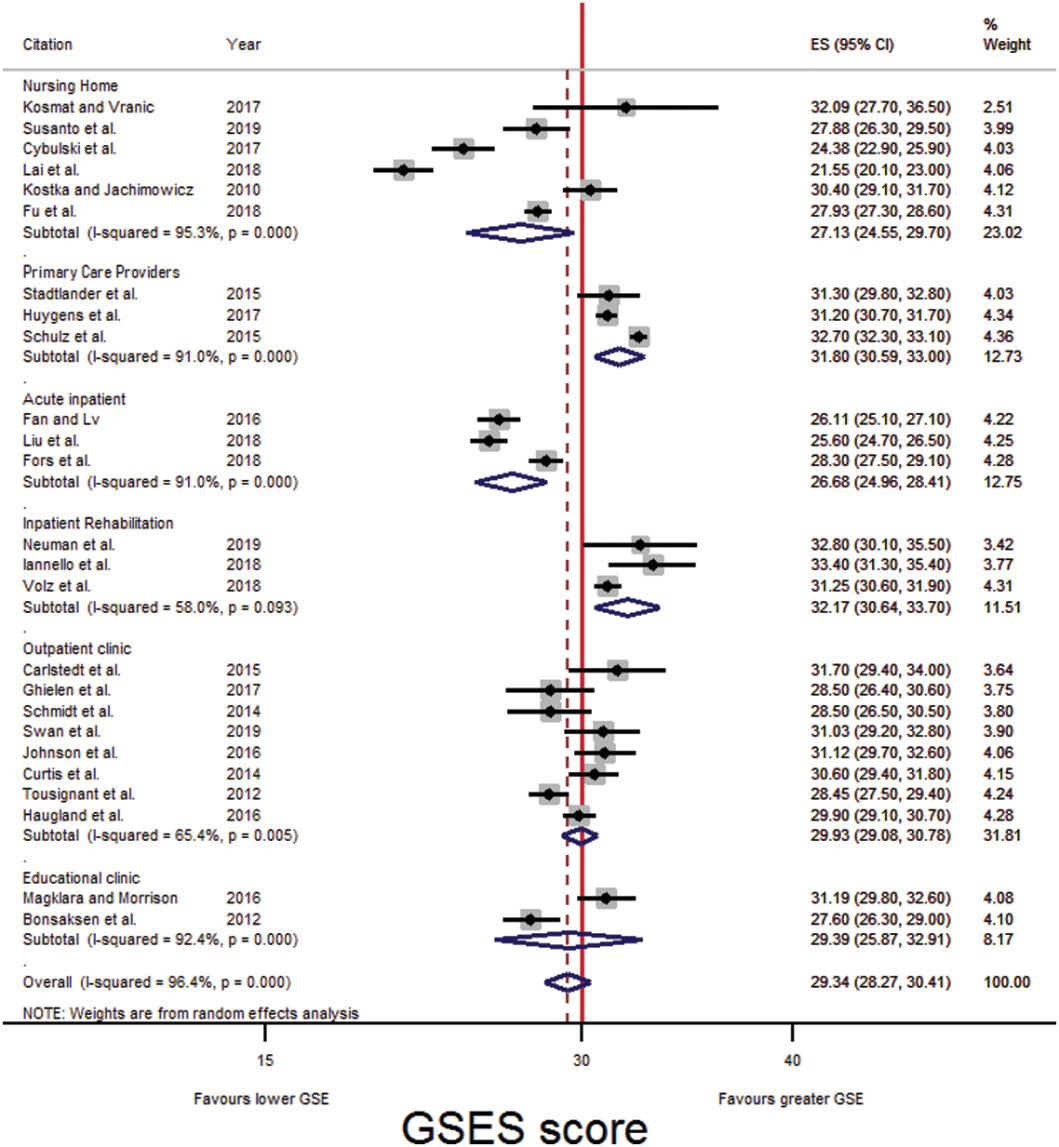
Forest plot: Comparison of GSES scores across six care settings, following leave-one-out analysis. CI = confidence interval; GSES = Generalized Self-Efficacy Scale.

Using the mean (with standard deviation [*SD*]) GSES score from individual studies, pooled mean GSES scores, and *SD*s were produced in Stata to compare the GSE of older adults across different health care settings (Figures 3–5). These “health care settings” were “inpatient care,” “outpatient care,” and “community care.”

Studies that recruited participants from inpatient wards, either acute medical or rehabilitative wards, were grouped together as “inpatient care.” Studies that recruited participants from outpatient clinics or educational clinics were grouped under “outpatient care.” Studies which recruited permanent residents of nursing homes were grouped under “community care” with studies concerning primary care providers (PCPs). This grouping of nursing homes reflects current literature regarding the provision of care within nursing homes: firstly, that any medical or rehabilitative care residents receive is primarily provided by community care services (e.g., community physiotherapists, or general practitioners) (Charles, 2019; Ghavarskhar, Matlabi, & Gharibi, 2018; Ribbe et al., 1997), and secondly, that nursing home residents are increasingly being seen as active members of communities (Tak, Kedia, Tongumpun, & Hong, 2015).

### Effect Size Estimations

The SMD measure of effect was used to compare the GSE of older adults in receipt of health care services with the GSE of older adults who were not receiving health care services (Figure 2). An SMD of zero would demonstrate that older adults receiving care, versus noncare, had comparable GSE. If the SMD value is negative, the results indicate that older adults without care have greater GSE. In this meta-analysis, the precision of the studies effect estimate determined the weight given to the SMD of each study.

To compare the GSE of older adults across health care settings, the mean GSES scores (with *SD*s) reported in each individual study were used to calculate pooled means and *SD*s for each care setting. Mean scores were weighted based on the precision of the studies estimate (the narrowness of the CI; Figures 3–5).

The literature search identified only one study which used the NGSES (Chen et al., 2001) and six which used the SES (Sherer et al., 1982); as a result, these were only included in the meta-analysis of SMD as there were not enough studies to calculate their pooled mean scores.

All meta-analyses were conducted in Stata (StataCorp, 2019).

### Missing Data

Eligible studies recruited “older adults,” which was determined by a mean age and lower 95% CI of at least 60 years old. The lower 95% CIs were calculated using the mean age and the *SD* of each sample, using the formula $\bar{x}\pm 1.96\left(\frac{\sigma }{\sqrt{n}}\right)$, where $\bar{x}$ is the sample mean, σ is the *SD*, and *n* is the sample size (Lane, 2020).

All but one study reported their samples age as a mean with the *SD*. Carlstedt, Lexell, Pessah-Rasmussen, and Iwarsson (2015) reported the mean age and the age range of their participants. To ensure that this study met the inclusion criteria, the *SD* of the sample mean was estimated using the range rule for *SD* ($\sigma \approx \;\frac{b-a}{4}$, where *a* is the minimum value and *b* is the maximum value [Ramirez & Cox, 2012]). Estimating the *SD* enabled the lower CI for the mean age to be estimated.

Eligible studies also had to report the GSE scale score of their participants. Mean GSE scale scores with standard deviations were required to carry out the meta-analyses. All the included studies provided these data; consequently, no further imputation of missing data was required.

### Assessment of Heterogeneity

For the meta-analysis of SMD, the I^2^ and chi-square statistics for heterogeneity were calculated. A random-effects model was applied given the clinical and methodological diversity across the included studies (Terrin et al., 2003).

### Sensitivity Analysis

Sensitivity analysis was performed using subgroup and leave-one-out analysis. Leave-one-out analysis is performed by omitting one study at a time to measure its individual effect on the pooled estimate of the rest of the studies (Viechtbauer & Cheung, 2010). This leave-one-out analysis also enabled the examination of outliers and influential statistics, thus identifying sources of heterogeneity.

### Outcomes

The search of online databases in September 2019 identified 18,706 publications. Following the exclusion of duplicates and the screening of titles and abstracts, 1,492 studies proceeded to full-text screening, a further 11 records were identified through manual searching of reference lists. Of these, 1,462 failed to meet the specified selection criteria, and one article was not accessible. The primary reason for exclusion was due to the assessment measure used; these primarily measured domain-specific self-efficacy (e.g., exercise self-efficacy). In total, 40 studies were eligible for this review (Figure 1).

### Study Characteristics

Study characteristics are reported in Table 2. Publication dates of the selected studies ranged from 2004 to 2019, with data from populations in the United States, Canada, Asia, Europe, and Australia. Studies included 33 different population cohorts, with sample sizes ranging from 19 to 1,018 participants and mean age between 63 and 88 years. One study recruited males only; in other studies, the proportion of males varied from 17.1% to 63.3%. Cross-sectional analysis was used in 23 studies, eight studies were randomized controlled trials (RCTs), three were cohort studies, four were pre–post studies with no control group; one study was a controlled, nonrandomized, pre–post study, and one followed a convergent mixed-methods design.

### Participants

A total of 4,731 participants receiving health care services were included in the review; of these, 49% received community care, 23.4% received inpatient care and 24% received outpatient care, and 3.6% of participants were described as “institutionalized” (Table 2).

### Self-Efficacy Measures

One study used the SES (Sherer & Adams, 1983), 38 studies used the GSES (Schwarzer & Jerusalem, 1995), and one study used the NGSES (Chen et al., 2001).

### Risk of Bias

Of the 40 studies, 25 were rated as having a “low risk of bias,” 12 were given a rating of “moderate risk of bias,” and three studies did not provide enough details to award a rating and so were categorized as having an “unclear” risk of bias. These ratings are given in Table 2.

The majority of the studies included in this review were of a cross-sectional design, most were deemed to have a “low risk of bias” due to high participation rates, use of defined recruitment criteria and standardized outcome measures, and controlling of potential cofounders. Due to the study design, there was also no loss to follow up. Studies that received “moderate risk of bias” ratings tended to not present discussion around its sample size, recruited less than 50% of eligible individuals or did not control for cofounders.

Within the cohort and pre–post studies, the greatest risk of bias came from loss to follow up. Percentages of loss ranged from <20% (Mystakidou et al., 2013; Volz, Möbus, Letsch, & Werheid, 2016) to > 50% (Bonsaksen, Fagermoen, & Lerdal, 2014; Neuman, Gaskins, & Montgomery, 2019). Studies accounted for loss to follow up through multiple imputation (Feldstain, Lebel, & Chasen, 2016), average imputation (Bonsaksen, Haukeland-Parker, Lerdal, & Fagermoen, 2013; Bonsaksen et al., 2014), and/or listwise deletion when data were deemed to be missing at random (Bonsaksen et al., 2013; Bonsaksen et al., 2014; Neuman et al., 2019). Volz, Voelkle, and Werheid (2018) adopted a continuous time perspective in which missing longitudinal data were translated into a problem of unequal time intervals.

Eight RCTs were included in this review; of these, six stated their study design and group characteristics in enough depth to determine that there was low risk of selection bias (Fors et al., 2018; Ghielen et al., 2017; Johnson, Booth, Currow, Lam, & Phillips, 2016; Lai et al., 2018; Swan, English, Allgar, Hart, & Johnson, 2019; Tousignant et al., 2012). The studies by Kosmat and Vranic (2017), Iannello and colleagues (2018), and Fors and colleagues (2018) were found to be of “unclear” risk of bias due to lack of detail regarding the randomization of participants and concealment of the groups.

With regards to performance bias, three RCTs did not blind their participants (Ghielen et al., 2017; Johnson et al., 2016; Swan et al., 2019), while four were unable to blind personnel as they were delivering the interventions (Johnson et al., 2016; Kosmat & Vranic, 2017; [Bibr CIT0083][Bibr CIT0086]). Regarding detection bias, only two studies blinded their outcome assessors (Lai et al., 2018; Tousignant et al., 2012). Finally, two studies did not report details regarding the blinding of either their participants or their outcome assessors (Fors et al., 2018; Iannello et al., 2018).

The RCTs are, therefore, at various risks of performance or detection bias. However, the use of functional performance measures, and measures that required the self-report of blinded participants, reduced the risk of bias in each study. This is similar in the quasi-experimental study by Strupeit, Wolf-Ostermann, Bu, and Dassen (2013). Additionally, the studies by Johnson and colleagues (2018) and Swan and colleagues (2019) were feasibility trials and so, the authors judged that their lack of blinding was not likely to influence the outcome of the studies as the source of bias would be consistent across study arms. Furthermore, the aim of the studies was to measure variability in response to measures to inform a further RCT.

Finally, the included convergent mixed-methods study (Stadtlander et al., 2015) was of appropriate design for its research aims and had a response rate of 100%. However, the sampling strategy resulted in few participants and the effect of the achieved sample size on the quantitative portions of the study was not discussed, increasing the risk of selection bias.

## Results

### The GSE of Older Adults and Receipt of Health Care Services

Five studies investigated the difference in GSE between a population of older adults receiving health care services, and a population of older adults who were not receiving care.

Pooling study effects demonstrated statistically significantly lower GSE in older adults receiving health care services than in older adults not receiving care (SMD = −0.62, CI: −0.96 to −0.27, *p* < .0001; *n* = 5, No. receiving care: 395, No. without care: 385; Figure 2).

### The GSE of Older Adults Across Different Health Care Settings

Thirty-one eligible studies used the GSES and published the mean scores of their participants (nine community, eight inpatients, and 14 outpatients; Figure 3); their GSES scores were pooled and forest plots produced. Reference lines were fixed at 30 as it is suggested that a GSES score of less than 30 is indicative of low self-efficacy (Haugland, Wahl, Hofoss, & DeVon, 2016).

Across all three settings, the pooled mean score was similar, being only very slightly higher in older adults receiving outpatient care (29.34 [28.19, 30.49]), compared with older adults receiving inpatient (28.83 [26.93, 30.74]) and community care (28.75 [26.66, 30.83]; Table 3).

**Table 3.** Comparison of GSES Scores Across Care Settings at Different Stages of Analysis

	Initial meta-analysis			Subgroup analysis		Post-leave-one-out analysis	
Setting	*N*	Mean (95% CI)	Setting	*n*	Mean (95% CI)	*n*	Mean (95% CI)
Community	2,381	28.75 (26.66, 30.83)	Nursing homes	701	27.13 (24.55, 29.70)	701	27.13 (24.55, 29.70)
			Primary care providers	1,680	31.80 (30.59, 33.00)	1,680	31.80 (30.59, 33.00)
Inpatient	1,106	28.83 (26.93, 30.74)	Acute medical	626	27.05 (25.61, 28.50)	527	26.68 (24.96, 28.41)
			Rehabilitation	480	30.77 (27.39, 34.15)	356	32.17 (30.64, 33.70)
Outpatient	1,087	29.34 (28.19, 30.49)	Clinic	948	29.33 (28.05, 30.61)	605	29.93 (29.08, 30.78)
			Education	139	29.39 (25.87, 32.91)	139	29.39 (25.87, 32.91)

*Note*: CI = confidence interval; GSES = Generalized Self-Efficacy Scale.

As studies had been grouped broadly, subgroup analysis was carried out. Six subgroups were formed, with studies grouped into those which recruited participants from nursing homes, PCPs, acute inpatient wards, inpatient rehabilitation wards, outpatient clinics, or educational courses.

Following subgroup analysis (Figure 4), older adults receiving care provided by PCPs had the greatest GSES score (31.80 [30.59, 33.00]), followed by inpatients in a rehabilitation ward (30.77 [27.39, 34.15], then those attending education courses (29.39 [25.87, 32.91]), then those attending an outpatient clinic (29.33 [28.05, 30.61]), followed by residents of nursing homes (27.13 [24.55, 29.70]), and lastly, those receiving acute medical inpatient care (27.05 [25.61, 28.50]; Table 3).

Heterogeneity was observed among the GSES scores in each subgroup. Leave-one-out analysis was performed to measure each study individual effect on the pooled estimate of the studies. Leave-one-out analysis could not be carried out with the educational course subgroup, due to only two studies being included.

Following leave-one-out analysis (Figure 5) older adults receiving inpatient care in a rehabilitation ward had the greatest GSES score (32.17 [30.64, 33.70]), followed by those attending their PCPs (31.80 [30.59, 33.00]), then those attending an outpatient clinic (29.93 [29.08, 30.78]), then those attending an educational course (29.39 [25.87, 32.91], then residents of nursing homes (27.13 [24.55, 29.70]), and lastly, those receiving acute medical inpatient care (26.68 [24.96, 28.41]; Table 3). Additionally, there was no overlap in 95% CIs for pooled GSES score between acute medical inpatient care and inpatient rehabilitation care, outpatient clinic care, or PCPs. Studies conducted in nursing homes and educational courses continued to demonstrate considerable heterogeneity.

## Discussion

The SMD highlighted a significant difference between the GSE scores of those receiving care and those who did not receive health care services. This supports the theory that GSE is contextual and may be influenced by the level and form of health care an older adult is receiving.

Additionally, our findings support Barder and colleagues (1994), who found that individuals receiving acute inpatient care services are at risk of having poorer GSE than those receiving community care.

While Haugland and colleagues (2016) suggest a GSES score of less than 30 is indicative of a low self-efficacy score of clinical significance, Schwarzer (2014) recommends that levels of self-efficacy are determined based on the empirical distributions of a particular reference population. In this analysis, the mean GSES score for all older adults, following leave-one-out analysis, was 29.34 (28.27, 30.41). In comparison, the GSES score of older adults receiving care was 27.13 (24.55, 29.70) in nursing homes, 31.80 (30.59, 33.00) in PCPs, 26.68 (24.96, 28.41) in acute inpatient settings, 32.17 (30.64, 33.70) in inpatient rehabilitation settings, 29.93 (29.08, 30.78) in outpatient clinics, and 29.39 (25.87, 32.91) in educational courses.

These findings suggest that following the experience of an unexpected admission to hospital, and increased reliance on health care professionals, older adults receiving inpatient care may perceive an increased inability to cope with and adapt to stressful life events; thus, reducing their GSE. Barder and colleagues (1994) support this finding as they concluded that individuals receiving acute inpatient care had reduced preference for control over health care than older adults receiving care in other settings. Furthermore, the results of Iannello and colleagues (2018) suggest that receiving inpatient care may reduce an older adults’ GSE, as their control group, who received standard inpatient care, had a reduction in GSE during their admission.

Recent research has investigated the relationships between demographic factors and older adults’ self-efficacy, suggesting that it is likely to be affected by factors such as age, relationship status, and education (Hur, 2018). The studies included in this review do not support this judgment. Several studies included in this review assessed the relationships between GSE and demographic factors, including age, gender, education, relationship status, and social support; only social support was found to be significantly related to GSE in over half of the studies it was investigated in (see Supplementary Material for the reported bivariate relationships between GSE and demographic variables).

Conducting leave-one-out analyses identified other potential factors that may influence the relationship between the health care setting and older adults’ GSE.

Firstly, the present review supports the premise that there is a relationship between illness severity, or illness perception, and GSE, as leave-one-out analysis identified that studies that recruited palliative care patients had lower GSES mean scores, and significantly increased the heterogeneity in the analysis. Moreover, the study by Mystakidou, Parpa, and colleagues (2010), which recruited patients receiving curative radiotherapy, was also found to be a source of heterogeneity and was also removed following leave-one-out analysis (Figure 4).

Considerable heterogeneity was also observed within the community-based studies. Conducting subgroup analysis highlighted the substantial variation in the GSE of nursing home residents. Though the reason for this is unclear, previous research has found that within nursing homes factors such as adaption to facility, decision to enter, the quality of care, length of stay, and social engagement influence the GSE of their residents (Chang, Park, & Sok, 2013; Choi & Sok 2015; Fu, Liang, An, & Zhao, 2018; Susanto, Rasny, Susumaningrum, Yunanto, & Nur, 2019). Nevertheless, these factors were not investigated consistently across the studies, and so the suggestion that they may contribute to the observed heterogeneity is speculative.

Previous research has also found that health care provision within nursing homes varies substantially across countries, with some including rehabilitative services (often those in the United States) while others have no, or very limited, access to rehabilitative services (e.g., in the United Kingdom, Denmark, Italy, China, and Australia) (Ghavarskhar et al., 2018; Ribbe et al., 1997). Given that this study found that older adults receiving inpatient rehabilitative care had greater GSE than those residing in nursing homes, it may be that individuals who receive rehabilitative services in nursing homes have higher GSE than those who do not. However, of the nursing home studies included in this review, only Susanto and colleagues (2019) mentioned that residents were receiving rehabilitative services, and their participants did not demonstrate higher GSE.

Finally, within the inpatient rehabilitation studies, the study by Strupeit and colleagues (2013) was found to be significantly heterogeneous. Unlike Iannello and colleagues (2018) and Neuman and colleagues (2019) who recruited participants following hip surgery, Strupeit and colleagues (2013) recruited participants with a diagnosis of functional mobility impairment or stroke. While Volz and colleagues (2018) also recruited stroke patients, their participants were approaching discharge, while Strupeit and colleagues (2013) recruited their participants shortly after they had been admitted. Strupeit and colleagues (2013) also recruited participants who resided either at home or at a nursing home. It is suggested, therefore, that the observed heterogeneity could also be explained by the illness perception of its participants or their place of residence.

### Limitations

This study is the first systematic review and meta-analysis to explore the differences in GSE between older adults receiving care in different health care settings. However, there are some limitations.

Firstly, GSE measures are used intermittently in research with a range of study designs, in various settings and with various population groups. As a result, analysis stratified by demographic or detailed clinical variables of participants was not prespecified, and observational study designs of reduced rigor were included. This limitation is highlighted in the substantial methodological heterogeneity between included studies; for this reason, no tests for heterogeneity were conducted between subgroups. Despite this, this review attempted to address the observed heterogeneity using a random-effects model, while subgroup and leave-one-out analyses were carried out to assess the robustness of the conclusions and to identify causes of heterogeneity (Higgins, 2008).

Secondly, imputation of data can decrease the certainty that can be placed in the results of this meta-analysis. However, only one study (Carlstedt, Lexell, Pessah-Rasmussen, & Iwarsson, 2015) included in this review required the imputation of data. Furthermore, it was for the 95% CI of the participants mean age and not data related to the GSES score. It is also deemed unlikely that the true 95% CI of the participants’ ages would have excluded this study from the review because the participants mean age was 68.1 (range: 58–86).

Lastly, language bias may also be considered as only studies that were published in the English language were selected; though the studies were conducted across a wide range of geographical locations, they comprised largely European populations.

### Clinical and Research Implications

GSE is an operative construct, that is, it is related to subsequent behavior and, therefore, is relevant for clinical practice and behavior change (Schwarzer, 1992). Considering the continued growth of the older population and given that GSE is predictive of positive health behaviors, it is of importance that GSE is considered and addressed in the care of older adults.

This systematic review and meta-analysis found that individuals receiving acute inpatient care are at risk of having lower GSE, in comparison with those in inpatient rehabilitation settings, attending outpatient clinics, or receiving PCP care. Additionally, the study by Iannello and colleagues (2018) suggests that older adults’ GSE may reduce during inpatient admission.

This finding should, however, be interpreted with caution, as the difference in findings of Volz and colleagues (2018) and Strupeit and colleagues (2013) suggest that GSE may increase as individuals approach to discharge. This finding is not dissimilar to the results of Tousignant and colleagues (2012), who found that while the GSE of their control group increased, after receiving standard day-hospital physiotherapy, a larger and longer-lasting improvement in GSE was seen in their experimental group, who received tailored tai-chi interventions instead. It is proposed, therefore, that even if GSE routinely increases closer to inpatient discharge, there is the potential for this to be enhanced.

Previous research has shown that interventions can be successful in improving the GSE of older adults (Jones et al., 2009; Tousignant et al., 2012). However, it is proposed that these interventions need to involve the active participation of the older adult, as a study that investigated the efficacy of increased nurse-led consultations following stroke rehabilitation found no significant differences in the final GSES scores of their intervention and control group (Strupeit et al., 2013). Furthermore, they should be based on everyday activities of older adults, or something that can be easily built into everyday life, as literature suggests that older adults need more tangible everyday experiences to bring about changes in subjective well-being (Enkvist, Ekström, & Elmståhl, 2012). GSE interventions differ from patient empowerment and engagement interventions, which are also being encouraged as a way to improve health, policy, and service delivery (World Health Organization [WHO], 2016).

Patient empowerment interventions often focus on education (WHO, 2016). Through education patients’ ability to act independently increases, encouraging them to engage in their own health management tasks (Khuntia, Yim, Tanniru, & Lim, 2017). In contrast, GSE interventions should focus on providing mastery experience, enabling patients to successfully complete tasks so that they feel more confident in attempting new behaviors (Köhler, Tingström, Jaarsma, & Nilsson, 2018). Successful engagement in health-related tasks may increase GSE; however, Köhler and colleagues (2018) warn that patient empowerment and GSE are not interchangeable, and that both need to be considered when planning health care provision.

Concerning older adults residing in nursing homes, the findings of this review suggest that they have the potential to have some of the highest and lowest levels of GSE among older adult populations. Nursing home managers should consider how they could foster their residents’ GSE because low GSE in nursing home residents is significantly related to both shorter life expectancy and greater death anxiety (Shokri & Akbari, 2016). The discussion has touched upon factors that have been found to influence the GSE of older adults residing in nursing homes; those that are modifiable should be considered as ways to improve residents’ GSE.

Considering the results of this review, we recommend that future research should focus on:

Firstly, the implementation and effectiveness of GSE interventions in inpatient care settings. Low GSE is understood to be a predictor of both negative health outcomes and poorer protective personality characteristics, such as resilience (Liu, Zhou, Zhang, & Zhou 2018; Stadtlander et al., 2015). As such, health care recommendations suggest that development of GSE-focused interventions will aid complex decision making in the healthcare of older adults (Hardy, Concato, & Gill, 2004; Hicks & Conner 2014; Kulakçi & Emiroǧlu 2013; Lee et al. 2013). Consequently, research is needed that investigates the relationships between GSE and other protective personality characteristics in older adults receiving inpatient care and their subsequent, postdischarge, health outcomes.

Finally, given that palliative care studies were found to be a large source of heterogeneity in this review, further research is needed to investigate whether the setting in which palliative care is given impacts upon the GSE of those receiving the care, and whether the setting could be altered to improve the quality of life of older adults approaching the end of their life.

## Supplementary Material

gnaa036_suppl_Supplementary_Material

## Notes

References marked with an asterisk indicate studies included in the meta-analysis.
