# Supplementary material for: The General Self-Efficacy of Older Adults Receiving Care: A Systematic Review and Meta-Analysis
Source: Gerontologist. 2020 May 6;61(6):e302–17. doi: 10.1093/geront/gnaa036 (PMC8361502; doi:10.1093/geront/gnaa036)
Supplement: gnaa036_suppl_Supplementary_Material [file gnaa036_suppl_supplementary_material.docx]

Supplementary resource 1:


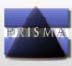
**PRISMA 2009 Checklist**

| **Section/topic** | **#** | **Checklist item** | **Reported in the section titled** |
| --- | --- | --- | --- |
| **TITLE** | | |  |
| Title | 1 | Identify the report as a systematic review, meta-analysis, or both. | Title |
| **ABSTRACT** | | |  |
| Structured summary | 2 | Provide a structured summary including, as applicable: background; objectives; data sources; study eligibility criteria, participants, and interventions; study appraisal and synthesis methods; results; limitations; conclusions and implications of key findings; systematic review registration number. | Abstract |
| **INTRODUCTION** | | |  |
| Rationale | 3 | Describe the rationale for the review in the context of what is already known. | Background |
| Objectives | 4 | Provide an explicit statement of questions being addressed with reference to participants, interventions, comparisons, outcomes, and study design (PICOS). | Background |
| **METHODS** | | |  |
| Protocol and registration | 5 | Indicate if a review protocol exists, if and where it can be accessed (e.g., Web address), and, if available, provide registration information including registration number. | Protocol and Registration |
| Eligibility criteria | 6 | Specify study characteristics (e.g., PICOS, length of follow-up) and report characteristics (e.g., years considered, language, publication status) used as criteria for eligibility, giving rationale. | Eligibility Criteria |
| Information sources | 7 | Describe all information sources (e.g., databases with dates of coverage, contact with study authors to identify additional studies) in the search and date last searched. | Search strategy |
| Search | 8 | Present full electronic search strategy for at least one database, including any limits used, such that it could be repeated. | Search strategy  Supplementary Material |
| Study selection | 9 | State the process for selecting studies (i.e., screening, eligibility, included in systematic review, and, if applicable, included in the meta-analysis). | Study selection |
| Data collection process | 10 | Describe method of data extraction from reports (e.g., piloted forms, independently, in duplicate) and any processes for obtaining and confirming data from investigators. | Data extraction |
| Data items | 11 | List and define all variables for which data were sought (e.g., PICOS, funding sources) and any assumptions and simplifications made. | Data extraction |
| Risk of bias in individual studies | 12 | Describe methods used for assessing risk of bias of individual studies (including specification of whether this was done at the study or outcome level), and how this information is to be used in any data synthesis. | Quality assessment  Table 1  Risk of bias |
| Summary measures | 13 | State the principal summary measures (e.g., risk ratio, difference in means). | Data synthesis |
| Synthesis of results | 14 | Describe the methods of handling data and combining results of studies, if done, including measures of consistency (e.g., I^2^) for each meta-analysis. | Data synthesis |
| Risk of bias across studies | 15 | Specify any assessment of risk of bias that may affect the cumulative evidence (e.g., publication bias, selective reporting within studies). | Quality assessment |
| Additional analyses | 16 | Describe methods of additional analyses (e.g., sensitivity or subgroup analyses, meta-regression), if done, indicating which were pre-specified. | Sensitivity analysis |

| **RESULTS** | | |  |
| --- | --- | --- | --- |
| Study selection | 17 | Give numbers of studies screened, assessed for eligibility, and included in the review, with reasons for exclusions at each stage, ideally with a flow diagram. | Outcomes  Figure 1 |
| Study characteristics | 18 | For each study, present characteristics for which data were extracted (e.g., study size, PICOS, follow-up period) and provide the citations. | Study characteristics  Table 2 |
| Risk of bias within studies | 19 | Present data on risk of bias of each study and, if available, any outcome level assessment (see item 12). | Risk of bias |
| Results of individual studies | 20 | For all outcomes considered (benefits or harms), present, for each study: (a) simple summary data for each intervention group (b) effect estimates and confidence intervals, ideally with a forest plot. | Results  Table 3  Figures 2-5 |
| Synthesis of results | 21 | Present results of each meta-analysis done, including confidence intervals and measures of consistency. | Results  Table 3  Figures 2-5 |
| Risk of bias across studies | 22 | Present results of any assessment of risk of bias across studies (see Item 15). | Results |
| Additional analysis | 23 | Give results of additional analyses, if done (e.g., sensitivity or subgroup analyses, meta-regression [see Item 16]). | Results: The effect of different healthcare settings on older adults’ GSE |
| **DISCUSSION** | | |  |
| Summary of evidence | 24 | Summarize the main findings including the strength of evidence for each main outcome; consider their relevance to key groups (e.g., healthcare providers, users, and policy makers). | Discussion |
| Limitations | 25 | Discuss limitations at study and outcome level (e.g., risk of bias), and at review-level (e.g., incomplete retrieval of identified research, reporting bias). | Limitations |
| Conclusions | 26 | Provide a general interpretation of the results in the context of other evidence, and implications for future research. | Clinical and Research Implications |
| **FUNDING** | | |  |
| Funding | 27 | Describe sources of funding for the systematic review and other support (e.g., supply of data); role of funders for the systematic review. | Funding |

*From:*  Moher D, Liberati A, Tetzlaff J, Altman DG, The PRISMA Group (2009). Preferred Reporting Items for Systematic Reviews and Meta-Analyses: The PRISMA Statement. PLoS Med 6(7): e1000097. doi:10.1371/journal.pmed1000097

Supplementary resource 2: Electronic search strategy for The Cumulative Index to Nursing and Allied Health Literature (CINAHL) database

| **CINAHL** |  |  |
| --- | --- | --- |
|  |  |  |
| Search no | Search term | Results |
| S1 | elder* | × |
| S2 | older people | × |
| S3 | MH "aged" | × |
| S4 | MH "aged, 80 and over" | × |
| S5 | geriatric* | × |
| S6 | S1 OR S2 OR S3 OR S4 OR S5 | × |
| S7 | MH "Self-efficacy" | × |
| S8 | Efficacy beliefs | × |
| S9 | “Subjective wellbeing” | × |
| S10 | Control | × |
| S11 | S7 OR S8 OR S9 OR S10 | × |
| S12 | MH "Hospitalization" | × |
| S13 | MH "Institutionalization" | × |
| S14 | Nursing Home | × |
| S15 | Health care setting | × |
| S16 | Residential | × |
| S17 | MH "Rehabilitation" | × |
| S18 | S12 OR S13 OR S14 OR S15 OR S16 OR S17 |  |
| S19 | S6 AND S11 AND S18 |  |
| S20 | S6 AND S11 |  |
|  | Total: |  |

Supplementary resource 3: Reported bivariate relationships between GSE and demographic variables.

|  |  | Age | Gender | Employment | Education | Comorbidity | Relationship  status | Social  support |
| --- | --- | --- | --- | --- | --- | --- | --- | --- |
| **OUTPATIENT CARE** | **EDUCATION COURSE** |  |  |  |  |  |  |  |
|  | **BONSAKSEN ET AL. (2012)** | ✗ | ✗ | ✗ | ✗ |  | ✗ | ✗ |
|  | **MAGKLARA AND MORRISON (2015)** | ✗ |  |  |  |  |  |  |
|  | **OUTPATIENT CARE** |  |  |  |  |  |  |  |
|  | **HAUGLAND ET AL. (2016)** | ✓* |  |  |  |  |  |  |
|  | **MYSTAKIDOU ET AL. (2010B)** | ✗ |  |  |  |  | ✗ |  |
|  | **MYSTAKIDOU ET AL. (2015)** | ✗ | ✗ |  | ✗ |  | ✗ |  |
| **INPATIENT CARE** | **ACUTE HOSPITAL** |  |  |  |  |  |  |  |
|  | **MYSTAKIDOU ET AL. (2010A)** | ✗ | ✓* |  |  |  | ✗ |  |
|  | **REHABILITATION** |  |  |  |  |  |  |  |
|  | **LEWIN ET AL. (2013)** | ✗ |  |  |  |  |  | ✓** |
|  | **STRUPEIT ET AL. (2013)** | ✗ | ✗ |  | ✗ |  |  |  |
| **COMMUNITY CARE** | **PRIMARY CARE** |  |  |  |  |  |  |  |
|  | **PAUKERT ET AL. (2010)** | ✗ |  |  |  | ✗ |  | ✓** |
|  | **STADTLANDER ET AL. (2015)** |  | ✓** |  | ✓* |  |  |  |
|  | **NURSING HOME** |  |  |  |  |  |  |  |
|  | **CYBULSKI ET AL. (2017)** | ✓*** |  |  |  |  |  |  |
|  | **FU ET AL. (2018)** |  |  |  |  |  |  | ✓** |
|  | **KOSMAT AND VRANIC (2017)** | ✗ |  |  |  |  |  |  |

* P<0.05; ** P<0.01; *** P<0.001
